# Mediational Effects of Self-Efficacy Dimensions in the Relationship between Knowledge of Dengue and Dengue Preventive Behaviour with Respect to Control of Dengue Outbreaks: A Structural Equation Model of a Cross-Sectional Survey

**DOI:** 10.1371/journal.pntd.0002401

**Published:** 2013-09-26

**Authors:** Affendi Isa, Yoon K. Loke, Jane R. Smith, Alexia Papageorgiou, Paul R. Hunter

**Affiliations:** 1 Norwich Medical School, University of East Anglia, Norwich, United Kingdom; 2 University of Exeter Medical School, Devon, United Kingdom; 3 St George's, University of London Medical School at University of Nicosia, Nicosia, Cyprus; Oswaldo Cruz Foundation, Brazil

## Abstract

**Background:**

Dengue fever is endemic in Malaysia, with frequent major outbreaks in urban areas. The major control strategy relies on health promotional campaigns aimed at encouraging people to reduce mosquito breeding sites close to people's homes. However, such campaigns have not always been 100% effective. The concept of self-efficacy is an area of increasing research interest in understanding how health promotion can be most effective. This paper reports on a study of the impact of self-efficacy on dengue knowledge and dengue preventive behaviour.

**Methods and Findings:**

We recruited 280 adults from 27 post-outbreak villages in the state of Terengganu, east coast of Malaysia. Measures of health promotion and educational intervention activities and types of communication during outbreak, level of dengue knowledge, level and strength of self-efficacy and dengue preventive behaviour were obtained via face-to-face interviews and questionnaires. A structural equation model was tested and fitted the data well (χ^2^ = 71.659, *df* = 40, *p* = 0.002, RMSEA = 0.053, CFI = 0.973, TLI = 0.963). Mass media, local contact and direct information-giving sessions significantly predicted level of knowledge of dengue. Level and strength of self-efficacy fully mediated the relationship between knowledge of dengue and dengue preventive behaviours. Strength of self-efficacy acted as partial mediator in the relationship between knowledge of dengue and dengue preventive behaviours.

**Conclusions:**

To control and prevent dengue outbreaks by behavioural measures, health promotion and educational interventions during outbreaks should now focus on those approaches that are most likely to increase the level and strength of self-efficacy.

## Author Summary

Dengue fever is one of the most rapidly increasing vector-borne diseases of humans in the tropics. There is currently no treatment and no vaccine, so control of the disease depends on controlling the mosquito vector. Unfortunately health promotional campaigns aimed at encouraging people to reduce mosquito breeding sites have not always been 100% effective. Self-efficacy is an area of increasing research interest and can be thought of as people's confidence in their ability to engage in health behaviours. We report a study of the impact of self-efficacy on dengue preventive behaviour. We conducted face to face interviews in villages in the state of Terengganu, Malaysia that had been affected by dengue outbreaks. A structural equation model was tested and fitted the data well. Mass media, local contact and direct information-giving sessions significantly predicted level of knowledge of dengue. However, self-efficacy fully mediated the relationship between knowledge of dengue and engagement in dengue preventive behaviours. We conclude that educational components of community dengue control programmes should focus on interventions.

## Introduction

Dengue fever is transmitted by the bite of an *Aedes* mosquito infected with any one of the four dengue viruses. Although most infections are self-limiting a proportion of cases develop severe complications such as dengue haemorrhagic fever which can carry a significant risk of death. The incidence of dengue has risen dramatically around the world in recent decades. Since no vaccine is currently available, primary prevention is regarded as the most effective measure in controlling dengue. Each time an outbreak occurs, the local health authority will plan and carry out various types of promotional and educational activities that aim to increase knowledge of dengue and change dengue preventive behaviour among communities at the centre of the outbreak. These promotional activities can be carried out through various methods such as individual home visits, or at the population level through the mass media. Health promotion and educational intervention like, ‘search and destroy’ activities, advice on the need to seek immediate medical attention in patients with fever, and proper disposal of rubbish are usually the focus of behavioural-change promotion activities. The promotional and educational messages are usually delivered using small group discussion, public lecture, live public announcement, demonstration, distributing printed materials, putting up posters, bunting and billboards, community source reduction and community dengue-cleanliness program (in Malay: *Gotong-Royong*) and health exhibition [1].

There have been a number of systematic reviews of public health interventions aimed at reducing the risk of dengue fever in recent years [2]–[6]. However, as pointed out by Bouzid et. al. authors have often reached different conclusions regarding the effectiveness of interventions, even when reviewing the same primary studies [7]. Health promotion campaigns that appear to have some benefit are those aimed at encouraging local people to engage in activities that reduce the number of mosquitoe breeding sites close to home [2]. However, such campaigns are not totally effective and the impact on vector presence may only be short-live. Achieving sustainable change in dengue preventive behaviours remains difficult [8], and may not necessarily lead to dengue prevention [9], [10], [11].

Dengue fever is endemic in Malaysia with frequent major outbreaks in the urban areas. Since dengue was first documented in Malaysia in 1902 and was made notifiable in 1973, the disease pattern has changed from major outbreaks every four years to one of increasing trend yearly. The largest outbreak was seen in 1996 with 14,255 dengue cases reported and 32 deaths. The fever is the number one disease in the top 10 listed communicable diseases in Malaysia as compared to other diseases like Tuberculosis, Malaria and HIV/AIDS in 2010 and 2011 [12]. The number of dengue cases reported also increased from 27,381 cases in 1998 to 46,171 cases in 2010. Estimate of an economic burden of dengue in Malaysia is USD102.25 (95%CI: 77.94–310.66) million per year which is approximately USD3.72 (95%CI: 2.83–11.30) per capita [12]. There is evidence that despite the fact that Malaysians generally have good knowledge of dengue fever and its prevention [13], dengue incidence rate has substantially increased from 31.6/100,000 population in year 2000 to 163/100,000 in year 2010 [12].

There is a growing body of literature concerning the concept of self-efficacy, which is considered to be people's belief or confidence in their capabilities to achieve different levels of performance attainment [14]. Self-efficacy perceptions are viewed as important determinants of behaviour and affect, and the potency of these perceptions in predicting behaviours in many domains has been shown [15]. The concept of self-efficacy is commonly used in studies of health behaviours [16], [17]. including area such as smoking cessation [18], [19], weight loss and body weight control [20]–[25], exercise [26], [27], [28], nutrition intake [29], [30], alcohol use [31]–[34], and AIDS prevention [35], [36], [37]. Self-efficacy may also function as a mediator between cognitions, feelings and behaviours and the adoption of lifestyle behaviours such as healthy diet [38]–[41]. Although the effects of health promotion and educational interventions to control dengue fever have been investigated in previous studies, none of the studies have investigated the impact of self-efficacy dimensions (level and strength of self-efficacy) as mediators between level of dengue knowledge and effective behavioural actions to control dengue outbreak and transmission. Strength of self-efficacy refers to a person's perceived assurance that they ‘can do’ or ‘cannot do’ something reflected in their affirmative answers to questions about whether they can perform particular dengue preventive behaviours. Level of self-efficacy is a person's judgement about whether or not they can accomplish a given performance which reflects their perceived capability as measured against task demands (dengue preventive behaviour) at various levels of challenge (scenarios) to successful control of dengue fever during outbreaks [42].

We argue that understanding the relationship between knowledge, self-efficacy and behavioural change may be a route towards improved and sustainable dengue control. This paper reports on work that was conducted to study the impact of self-efficacy on dengue preventive behaviours. We conducted a survey in villages that was subsequently examined with analyses based on predictions from Bandura's Social Cognitive Theory and Maibach's path model [42], [43]. We specifically examined the potential mediating effects that level and strength of self-efficacy may have on the relationship between knowledge of dengue and dengue preventive behaviour after being exposed to health promotion and educational interventions during the outbreaks.

## Methods

### Respondents

We recruited heads of families or their spouses aged above 18 years old from 27 villages that had recently experienced an outbreak of dengue fever. These villages were located in the state of Terengganu, on the east coast of peninsular Malaysia. Using the method by Woodward, we calculated that we needed a sample size of about 280 respondents [44]. This was based on a requirement to detect a Pearson correlation coefficient of 0.4 with a power of 80% and alpha 5% (200 samples). This sample size was then inflated by 20% to account for possible non-parametric tests and 20% for potential impact of clustering within village.

### Recruitment

**Figure 1:**
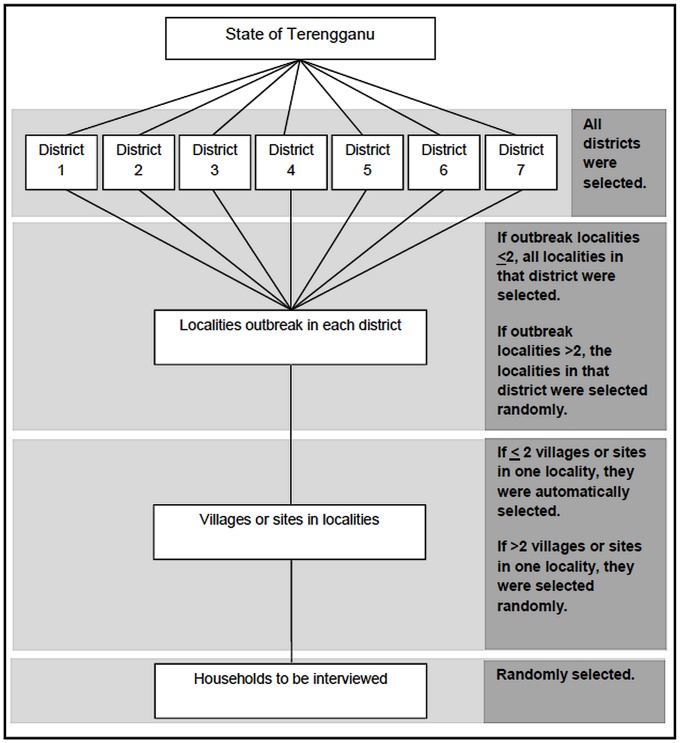
Summary of sampling procedures.

The population of the study included all the villages of the outbreak localities from July to December 2010 in the state of Terengganu. The list of outbreak sites was obtained from Terengganu Vector Borne Disease Division and the Terengganu Crisis Preparedness Resource Centre (CPRC) database. In total there were 32 outbreak locations for that 6-months period, but only 27 locations were included in the study as five others were not actually villages but higher education institutions and schools. Figure 1 shows a simplified illustration of sampling procedures used in this study. The households that were interviewed in those selected villages or sites were randomly selected based on the current outbreak list obtained from the Terengganu Crisis Preparedness Resource Centre (CPRC) database. The households were selected randomly from 9,959 houses or premises included in the study using SPSS. A total of 149 premises were excluded because they were non-owner premises or abandoned houses. The selected households were not changed or replaced with other households even if the first and second visits resulted in failure to meet some the participants for interview.

### Ethics statement

Research ethics approval for this study was granted by the National Medical Research Register, Ministry of Health Malaysia (NMRR-10-206-5412) and the Faculty of Health Research Ethics Committee of the University of East Anglia (2010/2011–13), the author's institution, prior to data collection. Written informed consent was obtained from all participants prior to completion of the survey.

### Data collection

The data collection was carried out from January to March 2011. The recruitment and training of the 32 interviewers from the local State Health Department staff was undertaken in December 2010. The interviewers were dedicated staff from the State Health Department whose usual tasks involved running health promotion and education activities during the outbreak. The training was conducted by the lead researcher assisted by the Head of the Health Promotion Unit, Terengganu Health State Department. All respondents were recruited after they had given informed consent. The interviewers read the questionnaires and the respondents gave their answers to those questions. The interviewers then ticked the answers in the column provided and recorded any subjective answers not listed in the answer scripts. A copy of the questionnaire is given in supplementary Text file S1. The questionnaire used in the field was in Malay. Correct translation was checked through a parallel back-translation by members of Malaysia Institute of Translation. The questionnaire was also tested in a pilot study in both languages.

### Measures of variables

#### 1. Health promotion and educational intervention exposure

Exposure to health promotion and educational interventions was assessed using sets of questions adapted from *Kriska's Modifiable Activity Questionnaire (MAQ)* [45], and the campaign exposure index developed by Maibach et. al. [42]. Even though the original concept of evaluation of the ‘intensity’ of the particular behaviour was unchanged, our questions were modified accordingly to suit the evaluation of the ‘intensity’ for the health promotion and education exposure. There were 3 main components assessed in this section, consisting of **involvement** in the health promotion and education activities, **sources** of information during the outbreak and **recent behaviour compliance** as promoted in the previous outbreak. For the details of respondents' **involvement** in the health promotion and educational activities, 1 mark was given for ‘Yes’ and no mark was given to a ‘No’. Respondents also were asked to list the types of activities in which they were involved and they were given marks ranging from 1 to 5 accordingly, with the highest possible mark being 15 if they were involved in all the activities. The respondents were then asked about the number of days of their involvement in the activities. The answer ranged from 0 (minimum) to 14 (maximum) in line with the need for an outbreak to be controlled within 14 days. These answers were later converted to a categorical scale according to Health Promotion Intervention Guidelines during Dengue Outbreaks, Ministry of Health Malaysia [1] as shown in table S1 in Text S2.

For respondents' scores on the sources of information on dengue prevention during the outbreaks, 1 mark was given for ‘Yes’ and 0 mark for ‘No’. The types of information sources the respondents received were scored between 1 to 6 marks, the maximum score being 20 if they received the information from all the sources listed. Respondents also were asked about types of health promotion and education activities they were involved in and the duration of their involvement. The marks for sources of information and types of activities involved were given based on its priority as indicated in the guidelines [1]. Assessment of respondents' recent behaviour compliance was determined by the number of days they performed ‘search and destroy’ activities by themselves and the duration of their engagement. The number of days recorded ranged from 0 and 2 days only because they were taught to perform the activity once a week for a minimum of 20 minutes per session. The overall health promotion and educational intervention exposures weighted score was measured as in table S2 in . The total mark determined the exposure level of the respondents to the health promotion and education during the dengue outbreak based on Maibach's index of health campaign exposures [42].

#### 2. Health promotion and educational intervention activities and sources of information

When constructing the model, there were 11 health promotion activities or sources of information that could affect knowledge of dengue. Several of these activities or sources of information were highly correlated and so we used factors instead of the original variables. Factor analysis was done in SPSS using principal component extraction with Equamax rotation. Factor scores, derived by regression, were then saved for use in the Structural Equation Model.

#### 3. Dengue preventive behaviour

The indication for behavioural compliance and maintenance was measured through systematic calculation of recent behaviour of the respondents. This calculation was based on the number of days that the respondents performed ‘search and destroy’ activity within the last 14 days, and the duration of the activity for each session. These types of behaviour were taken into consideration in the analysis since they were the main aspects that should have been modified and maintained as promoted during the dengue outbreak interventions. The measurement of such dengue preventive behaviours during outbreak was adopted based an additive index measuring behavioural occurrences per week by Maibach et. al. [42]. The measurement was in weighted scores in which the maximum total scores were 20.

#### 4. Knowledge of dengue

The level of knowledge was assessed using a set of questionnaires adopted from the Malaysia Health Morbidity Survey [46]. The respondents were asked to choose the correct answers based on their knowledge of dengue fever, its signs and symptoms, indoor and outdoor breeding sites and specific dengue preventive behaviour measures to control dengue fever transmission. The questions were arranged according to Bloom's Taxonomy of Educational Objectives [47]. One mark was given for each correct answer. The maximum total mark was 25 and this was transformed into a percentage, as in table S3 in Text S2, to indicate the level of knowledge on dengue from poor to excellent based on 3 educational goals of Bloom's Taxonomy of Educational Objectives [47].

#### 5. Self-efficacy dimensions

Level and strength of self-efficacy were measured using Likert Scales from 1 (Not at all confident) to 10 (Extremely confident) adopted from Maibach et. al. [42]. Level of self-efficacy was measured through several specific scenarios related to behavioural confidence to prevent dengue transmission, while strength of self-efficacy was determined by three graded questions that ranged from simple to more complex. The behavioural scenarios themselves were graded to assess self-efficacy in increasingly difficult situations. The level and strength of the self-efficacy measures were derived from the mean scales of all answers and subsequently categorized into 5 groups based on 5-point mean measurement of Likert Scales, as in table S4 in Text S2, [48]. Results from Confirmatory Factor Analysis (CFA) on each of pooled items for level and strength of self-efficacy yielded factor loadings ranged from 0.72 to 0.99. Therefore, because of pooled factor loadings >0.5 we retained all of the items for further analysis as suggested by Hair et. al. [49].

### Statistical analyses

Pearson or Spearman correlation coefficients used in analyses between the three main outcome measures: exposure to health promotion and education, knowledge of dengue and self-efficacy dimensions. Regression modeling was carried out using Generalized Estimating Equations of SPSS 18 to account for cluster sampling at the village level. Based on consideration of Bandura's theory, the research model by Maibach et al. [42], and previous empirical findings on related public health issues and significant correlations from the investigation, an initial proposed model was constructed [38]–[41]. A structural equation model (SEM) was developed using AMOS version 18 [50]. The model reflects the relationships between variables obtained in the study in order to predict the dengue preventive behaviour change resulting from an increased dengue knowledge level and self-efficacy dimensions after being exposed to health promotion and educational intervention during the outbreak. SEM was used to test the proposed model against the observed dataset. SEM is a combination of factor analysis and path analysis and it is a confirmatory rather than an exploratory technique, because it compares a hypothesized model's covariance matrix with that of the observed data. Since the proposed model of this study involved observed variables, SEM allows us to determine significant paths between those variables in deriving a better explanation of their significant relationship findings based on the research hypotheses and proposed model.

There are several steps in analysing SEM using AMOS: 1) to develop a model based on research theory; 2) identify unique values that can be used for the parameters to be estimated in the proposed model; 3) apply various estimation techniques, for example in this study, maximum likelihood; and 4) test the fit of the model against the data. According to the results, the researcher might 5) modify the measurement model based on theoretical justifications; revise the model by adding, deleting, or modifying relationships between variables; or use measures indicating lack of fit for specific parts of the model when theoretically justified in the Modification Indices table [51]. Goodness of fit indices were used as indicators of model fit. Chi-square tests were used as an index of the significance of the discrepancy between the original (sample) correlation matrix and the (population) correlation matrix estimated from the model. Because the significance of chi-square tests is dependent on the number of subjects, the comparative fit index (CFI) and the root mean square error approximation (RMSEA) were further considered. CFI values are derived from the comparison of the hypothesized model with the independence model. RMSEA values help to answer the question of how well the model with unknown but optimally chosen parameter values would fit the population covariance matrix if it were available [52]. The lower the discrepancy measured by the RMSEA the better, with an RMSEA of 0.0 indicating a perfect fit. Acceptable values are CFI>.90 and RMSEA<.08.

Once the model fitted the data well, the next step was to test the mediation effect of self-efficacy dimensions on the relationship between knowledge of dengue and dengue preventive behaviour by comparing a Full Mediation Model, Direct Model and Indirect Model as recommended by Baron and Kenny [53], and Hayes [54]. The post-hoc probing test for mediation effect significance was performed to determine if the drop in the total effect (i.e. level of dengue knowledge to dengue preventive behaviour) was significant upon inclusion of mediator (level or strength of self-efficacy) in the model [55].

### Hypotheses

We aimed to test two primary hypotheses, namely (i) knowledge of dengue is directly associated with dengue preventive behaviours and (ii) both strength and level of self-efficacy are associated with dengue preventive behaviour.

## Results

### Descriptive analysis

We recruited 280 participants as per the sample size calculation. More than half of the respondents were female (58.9%). Their mean age was 42.7 years, and the majority (57.5%) were aged between 36 to 55 years old, and were married (96.1%). The ethnic background of the respondents was 98.6% Malay with the remainder being Chinese. Nearly half of the respondents were housewives (45%).

Table 1 presents the means, standard deviations, percentiles and ranges for all the principle scores. The level of health promotion and educational intervention exposure was low, with only 20% of the respondents receiving a moderate to high level of exposure. The respondents seemed to have moderate (40.7%) to good (38.6%) knowledge of dengue. In general, the self-efficacy of the respondents was at the moderate level. Although it was also found that 62.2% of respondents perceived they were relatively confident in performing dengue preventive behaviours, only 1.1% of them reported having excellent strength of self-efficacy. About half of respondents (45.4%) showed moderate levels of self-efficacy, while 36.4% had little confidence and felt uncertain how to perform these kinds of dengue preventive behaviours. 2.5% of them reported below the average confidence (mean = 2.99).

**Table 1:** Means, standard deviations, 25^th^ percentiles, median, 75^th^ percentiles and ranges for study variables.

Variables	*M* (*SD*)	25^th^ %ile	Median	75^th^ %ile	Range
Health Promotion and Educational Intervention Exposures Score	27.53(18.45)	15	25	36	0–96
Knowledge Score	17.38 (5.25)	13	17	22	4–25
Level of Self-Efficacy Scale	5.55 (1.50)	4.55	5.44	6.55	4.5–6.5
Strength of Self-Efficacy Scale	5.65 (1.42)	4.60	5.40	6.60	2.8–9.6
Dengue Preventive Behaviours Score	10 (6.693)	5.00	10.00	10.00	0–20

Most of respondents said they had received health information on dengue fever from Public Announcements (57.5%), Television (57.9%) and the Newspaper (44.6%). In term of respondents' participation in the health promotion and educational interventions, most of them tended to be involved in the Community Source Reduction Program or *Gotong-Royong* (60%) as compared to the Public Lecture (24.3%). Only 4.6% were involved in Demonstration activities. Regarding respondents' recent behaviour to control dengue outbreak and transmission, 73.2% of them failed to perform a 10-minute search and destroy exercise to eradicate *Aedes* mosquitoes breeding sites within the last 14 days.

With regards to dengue preventive behaviour, about half (45.5%) of the respondents did not comply with correct behaviours to control dengue fever transmission. Moreover, 30.4% of them had carried out only 5 minutes of cleanliness activity within the past 14 days. Only 23.9% of the respondents were found to comply with the correct behaviours to prevent dengue fever transmission as promoted in the educational interventions during the outbreaks.

### Bivariate analysis

Four factors were extracted from the data on information sources and together these four factors represented 60.1% of the variance in the original variables. Table 2 shows the rotated component. Factor 1 was associated with obtaining information through Television, Radio and Newspaper (regression score >0.5). We named this factor Mass Media. Factor 2 was associated with participation in *Gotong-royong* and obtaining information from public announcements and outdoor media. This factor was named Local Contact. Factor 3 was named Small Group Contact (Small Group Discussion and Demonstration) and Factor 4 was named Direct Information-Giving Session (Public Lecture and Individual Advice).

**Table 2:** Rotated Component Matrix showing the correlation between the first 4 factors and reporting of participation in health education activity or source of information.

	Component			
	1	2	3	4
HE Activity (Public Lecture)	−.114	.423	.006	.686
HE Activity (*Gotong-Royong*)	.159	.542	.100	.208
HE Activity (Individual Advice)	.309	−.085	.389	.615
HE Activity (Small Group Discussion)	.050	.051	.768	.214
HE Activity (Demonstration)	−.010	.146	.815	−.076
Source of information (Public Announcements)	.002	.706	.124	.007
Source of information (Outdoor Media)	.257	.737	.043	.021
Source of information (TV)	.720	.027	−.008	.264
Source of information (Printed Media)	.407	.402	.201	−.415
Source of information (Radio)	.766	.239	.093	−.060
Source of information (Newspaper)	.821	.133	.053	−.083

A correlation matrix was generated that included each of the variables in the study (see Table 3). Overall, the bivariate relationships between the majority of independent and dependent variables were weak. The relationship between Factor 1 from the health promotion and educational intervention (Mass Media) and mean dengue knowledge scores was the strongest (r = 0.326, *p*<0.01) as compared to other factors. Significant bivariate relationships were evident between dengue knowledge and level (r = 0.262) and strength of self-efficacy (r = 0.363) at *p*<0.01. Level of self-efficacy was significantly correlated with strength of self-efficacy (r = 0.383) at *p*<0.01 and dengue preventive behaviour (r = 0.212) at *p*<0.05. There was a significant correlation between dengue knowledge and dengue preventive behaviour. There was no significant difference in level of dengue knowledge between those respondents who were exposed to different levels of health promotion and educational intervention. However, there were different degrees of strength in self-efficacy among those who were exposed to different levels of health promotion and educational interventions (*p* = 0.022). The level of self-efficacy however was no different among them. Although not included in the SEM, we found a significant correlation between proportions of villages who didn't undertake at least 10-minutes-cleanliness behaviour per week with the duration of the outbreak in the village (*p* = 0.044).

**Table 3:** Correlation matrix between dengue knowledge, level and strength of self-efficacy and dengue preventative behaviours.

	HP&E Exposures	Knowledge Level	Level of Self-Efficacy	Strength of Self-Efficacy
Knowledge Level	0.306**			
Level of Self-Efficacy	0.173**	0.262**		
Strength of Self-Efficacy	0.219**	0.363**	0.383**	
Dengue Preventive Behaviours		0.112**	0.212*	0.147*

HP&E = Health Promotion and educational intervention.

* Significant at the .05 level.

** Significant at the .01 level.

### Path analysis

Examination of our proposed model using SEM of AMOS 18 indicated that adjustment could be made to improve the match between the data and model (χ^2^ = 75.622, df = 41, p = 0.189, CFI = 0.870, TLI = 0.895, RMSEA = 0.098). To identify the sources of error in the proposed model as indicated in Modification Indices, we eliminated paths that were not significant one at a time in order to find the most parsimonious model. First we eliminated the path between Factor 3 from the health promotion and educational intervention (small group contact) and knowledge of dengue. Second, we dropped the path between knowledge of dengue and dengue preventive behaviours. Table 4 contains the models' goodness of fit indices.

**Table 4:** Goodness of fit Indices for structural equation model of reporting dengue preventive behaviours.

Model	χ^2^	*df*	*p*	CFI	TLI	RMSEA
Proposed	70.835	39	0.005	0.892	0.895	0.095
Remove regression path of Factor 3 from Health Promotion and Educational Intervention to knowledge on dengue	71.659	40	0.003	0.916	0.927	0.089
Remove regression path of knowledge on dengue to dengue preventive behaviours	73.795	41	0.002	0.972	0.963	0.054

**Figure 2:**
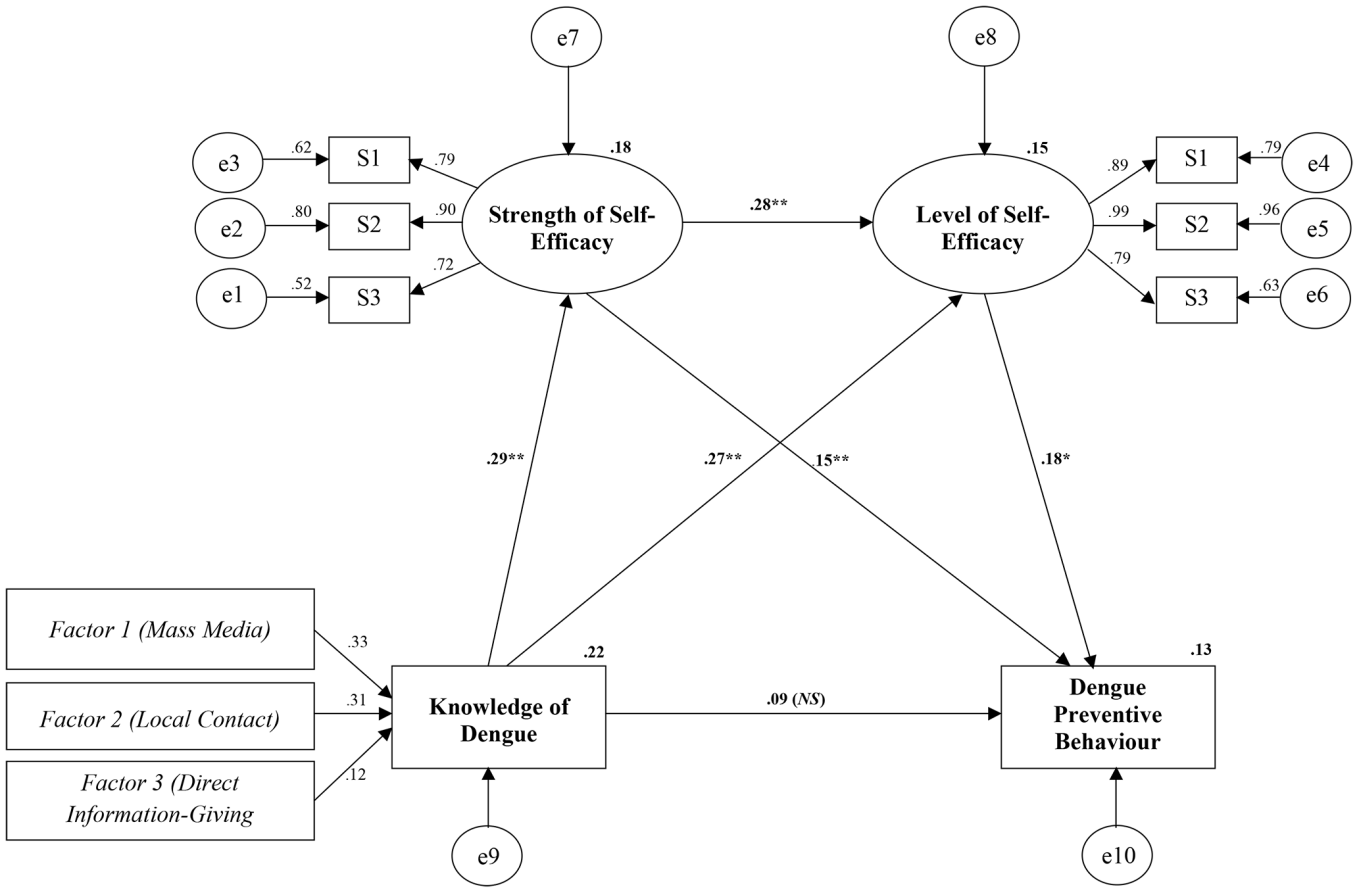
Final structural model of factors influencing. ** Significant at the .01 level. * Significant at the .05 level. (NS) Not significant. SE = Self-Efficacy, Factor 1 = Mass Media (TV, Radio, Newspaper), Factor 2 = Local Contact (*Gotong-royong*, Public Announcement, Outdoor Media), Factor 3 = Small Group Contact (Small Group Discussion, Demonstration), Factor 4 = Direct Information-Giving Session (Public Lecture, Individual Advice), L1 = Pooled Level of Self-efficacy (Scene 1), L2 = Pooled Level of Self-efficacy (Scene 2), L3 = Pooled Level of Self-efficacy (Scene 3), S1 = Pooled Strength of Self-efficacy (Personal Domain), S2 = Pooled Strength of Self-efficacy (Family Domain), S3 = Pooled Strength of Self-efficacy (Community Domain).

Our final model fitted the data well (χ^2^ = 71.659, *df* = 40, *p* = 0.002, CFI = 0.973, TLI = 0.963, RMSEA = 0.053). Bentler [56], and Chou [57] both recommend CFI and TLI scores of greater than 0.90 as indicators of good fitting models. Browne & Cudeck [58], (1993) and Byrne [52] recommend that models with an RMSEA of 0.08 or less and preferably 0.05 or less are good fitting models. Figure 2 shows the entire final model with accompanying path coefficients. Overall, the structural model contains relatively weak influences on the dengue preventive behaviours, with path coefficients ranging from 0.092 to 0.271.

### Mediation effect analysis

Our main objective was to investigate the self-efficacy dimensions as mediators of the relationship between dengue knowledge and dengue preventive behaviours in relation to control of dengue outbreaks. Assessment of the mediation effects was done by comparing the full mediation model (which includes a direct model) and indirect model from the final structural model that we created earlier. Table 5 shows the mediation effect findings of this models comparison.

**Table 5:** Model mediation effect findings for structural equation model of reporting dengue preventive behaviours.

Hypothesized Path	B (Estimate)	SE	Beta	CR	*p*
***Direct Model 1***					
Knowledge→Behaviour	1.610	0.756	0.092	2.128	0.082
***Direct Model 2***					
Level of SE→Behaviour	0.555	0.294	0.179	1.886	0.036
***Full Mediation Model 1***					
Knowledge→Level of SE	0.653	0.155	0.272	4.227	<0.001
Level of SE→Behaviour	0.370	0.312	0.179	1.184	0.036
Knowledge→Behaviour	1.141	0.804	0.090	1.420	0.093
***Full Mediation Model 2***					
Knowledge→Strength of SE	0.679	0.181	0.291	3.751	<0.001
Strength of SE→Behaviour	0.266	0.301	0.149	0.882	<0.001
Knowledge→Behaviour	1.141	0.804	0.090	1.420	0.093
***Full Mediation Model 3***					
Strength of SE→Level of SE	0.266	0.071	0.282	3.768	<0.001
Strength of SE→Behaviour	0.266	0.301	0.149	0.882	<0.001
Level of SE→Behaviour	1.370	0.312	0.178	1.184	0.032

From the initial analysis, knowledge of dengue did not have a direct effect on dengue preventive behaviour (standardized β weight = 0.092, *p* = 0.082). However, knowledge significantly predicted the level of self-efficacy as expected (standardized β weight = 0.172, *p*<0.001), and this level of self-efficacy also significantly predicted dengue preventive behaviour (standardized β weight = 0.179, *p* = 0.036). Knowledge had a direct effect on strength of self-efficacy (standardized β weight = 0.291, *p*<0.001) and this strength of self-efficacy also significantly predicted dengue preventive behaviours (standardized β weight = 0.149, *p*<0.001). Analysis for model comparison as recommended by Baron and Kenny [53], and Hayes [54] found that the *Beta* for the Indirect Model was reduced from 0.092 to 0.090 in the Full Mediation Model (in both for level of self-efficacy and strength of self-efficacy as mediators). Therefore, knowledge on dengue was found to have significant **indirect effect** on dengue preventive behaviour with a **mediation effect** of level of self-efficacy or strength of self-efficacy on the relationship.

Post-hoc probing of significant mediation effects was performed using the Sobel Equation of computing to determine if the drop in the total effect (i.e., knowledge on dengue) is significantly dependent upon inclusion of the mediator (level of self-efficacy and strength of self-efficacy) in the model [53], [55], [59]–[60]. This strategy indicated that level of self-efficacy (*z* = 4.77, *p*<0.05) and strength of self-efficacy (*z* = 2.38, *p*<0.05) did function as mediators. According to Holmbeck [55], *p*<0.05 is the absolute value of *z*>1.96. In addition since the *Beta* for the total effect of the relationship between knowledge on dengue and dengue preventive behaviours was 0.37, thus roughly 65% of the path was accounted for by level of self-efficacy as a mediator (*Beta* for indirect effect was 0.2405). Likewise, the path between knowledge of dengue and dengue preventive behaviours was 97% accounted for by strength of self-efficacy as a mediator in the relationship (*Beta* for indirect effect was 0.3575) [61]. Therefore, since the direct path between knowledge on dengue and dengue preventive behaviours was not significant, level and strength of self-efficacy did function as full mediators of that relationship. This result showed that self-efficacy has a complete mediation effect on the relationship between knowledge on dengue and dengue preventive behaviour.

As we hypothesized, strength of self-efficacy and level of self-efficacy significantly predicted dengue preventive behaviours (*p*<0.001). In addition, strength of self-efficacy significantly predicted level of self-efficacy (standardized β weight = 0.282, *p*<0.001). Later analysis for models comparison found that, the *Beta* for the Indirect Model was reduced from 0.179 to 0.168 in the Full Mediation Model. Therefore, strength of self-efficacy was also found to have a significant **indirect effect** on dengue preventive behaviour with a **mediation effect** of level of self-efficacy on the relationship.

Once again, post-hoc probing of significant mediation effects was performed using the Sobel equation of computing as a follow-up to the findings for the structural equation model. The post-hoc strategy was conducted to determine if the drop in the total effect (i.e., strength of self-efficacy) is still significant upon inclusion of the mediator (level of self-efficacy) in the model. This strategy indicated that level of self-efficacy did function as mediator (*z* = 2.020, *p*<0.05). (Note that *p*<0.05 if the absolute value of *z*>1.96). In addition since the *Beta* for the total effect of the relationship between strength of self-efficacy and dengue preventive behaviours was 0.3695, thus roughly 43% of the path was accounted for by level of self-efficacy as a mediator (*Beta* for indirect effect was 0.1596) [61]. In this case, level of self-efficacy **partially mediated** the association between strength of self-efficacy and dengue preventive behaviours. This result showed that level of self-efficacy has a partial mediation effect on the relationship between strength of self-efficacy and dengue preventive behaviour.

## Discussion

This investigation is one of the first within the public health and health psychology research literature to concentrate on health promotion and educational interventions designed to reduce the risk of dengue fever. With regards to our first primary hypothesis, knowledge was found not to be independently associated with dengue preventive behaviour other than through the impact of knowledge on self-efficacy. For the second hypothesis, both level and strength of self-efficacy were predictive of dengue preventive behaviours. Our hypothesis regarding the mediational effect of self-efficacy on the relationship between knowledge on dengue and dengue preventive behaviours was supported through post-hoc probing, as recommended by Holmbeck [55]. Thus, our work would suggest that increasing the public's knowledge about dengue fever is an essential first step towards encouraging people to engage in dengue preventive behaviours. However, increasing knowledge alone would not be sufficient unless it results in increasing the level and/or strength of people's confidence in performing these behaviours. These findings are consistent with previous work on self-efficacy and healthy lifestyle behaviours [15], [38]–[41]. Our findings hold a number of important implications for health promotion authorities and planners. This is because, since both self-efficacy level and strength are modifiable and reliable mediators of health behaviours [62], health promoters should design dengue educational interventions and campaigns that promote self-efficacy as well as knowledge.

With regards to knowledge generation, we have shown that mass media campaigns such as TV, radio and newspapers and local contact (*Gotong-royong*, public announcements, and outdoor media) and direct face-to-face communication session (public lecture and individual advice) are the most effective. For self-efficacy, various authors have suggested that one of the most effective means of promoting self-efficacy is through modelling socially relevant enactments of the behaviours in the mass media [15], [42], [62]–[65]. For example, in order to empower people to perform dengue preventive behaviours, the health authorities could produce video-taped material with trained role-players/actors performing the desired behaviours. The specially made videos could be shown to people who do not feel confident performing the specific preventive behaviours. People could then be asked to perform the desired behaviours until a level of competence and confidence was achieved. These activities could be promoted in groups or at individual level.

We would also add that since the self-efficacy dimensions are a cognitive response to direct and vicarious experiences with the behaviours, health promotion and educational interventions should use persuasive messages on dengue prevention. This is to enable the community to translate the messages into the anticipated or actual dengue prevention behaviour (example of a persuasive message: “If it breeds, we bleed, take action! Only 10 minutes to destroy *Aedes* breeding sites”). This accords with Bandura's idea on health campaign messages that successfully encourage the target audience to engage in simply enacted interim behaviour which will serve to enhance self-efficacy through direct experience. In relation to dengue prevention, such interim behaviour might include both trial performances of the behaviour such as not purchasing flower pots that accumulate water, as well as low-level versions of the target dengue preventive behaviour such as putting garbage in a closed bin instead of accumulating it in a group for incineration. Tailored messages tend to be more personally relevant and thus attract more attention [66]. When recipients received messages tailored to their personal information processing style, they were later more likely to engage in the desired behaviour advocated in the message [67]–[69].

Therefore, in health promotion and educational interventions during dengue outbreaks, guidelines and protocols should outline vividly the specific persuasive messages to be conveyed to specific target audiences during the outbreaks in order to increase self-efficacy dimensions. The health promotion and educational interventions should advocate dengue preventive behaviour that takes account of the psychological characteristics of the desired behaviour and of the information-processing style of the target population. This simple strategy may lead to better crafting of persuasive messages, which in turn, increase adoption of dengue preventive behaviour so that outbreaks and transmission are reduced.

In considering the generalizability of our study it should be noted that our respondents were predominantly based in rural villages that had recently experienced a dengue outbreak. Our findings may not be applicable in areas where there had been no prior experience of dengue fever. However, given the fact that the vast majority of the world's currently at risk population will have had prior experience, our findings ought to remain applicable. Clearly our study was conducted in a rural Malaysian population and so there are issues about whether the results can be generalised to urban populations or to rural populations in other countries with different cultures. It should also be noted that because the structural equation model was based on cross-sectional data, there should be some degree of caution on the interpretation of causal inferences. Nevertheless, it should be noted that these causal paths were hypothesized based on available research concerning predictors of dengue preventive behaviours during the outbreaks [12], [70], [71]. There remains an issue regarding possible reporting bias in the data collection, especially as the interviewers were also involved in the health promotion activities. The importance of not leading the respondents in any questions was stressed during the interviewers training sessions in order to minimise this source of bias. Although we cannot say definitively that there was no bias in our data collection, we would argue that the complexity of the model and the relationships between self-efficacy and knowledge independent of any particular health promotion activity would suggest that interviewer bias would have been unlikely to have played a major role in the main findings of this study.

Our findings address the important need for studies that generate empirically sound and theoretically relevant data to identify variables likely to be effective for designing interventions. Further research should aim to describe other aspects of psychological variables related to behaviour changes and maintenance in relation to control of dengue fever, such as the complex role of motivation as well as perceived barriers and perceived benefits to engaging in the target behaviours [72]–[76]. Furthermore, we should also consider future intervention studies that evaluate different mediation effects of level and strength of self-efficacy as separate psychological components in predicting other health behaviours to control or prevent public health diseases.

In conclusion, our research indicates that the impact of public health campaigns designed to increase the adoption of behaviours by the public to control dengue fever by increasing knowledge is mediated by the impact on self-efficacy. We argue that to be most effective public health campaigns should be designed to maximise the impact on self-efficacy. There is a strong need for further research on how to design public health campaigns for the control of vector-borne disease that maximise self-efficacy and not just knowledge.

## Supporting Information

Text S1 Questionnaire.
(PDF)

Text S2 Additional tables.
(PDF)
