# Supplementary material for: Mediational Effects of Self-Efficacy Dimensions in the Relationship between Knowledge of Dengue and Dengue Preventive Behaviour with Respect to Control of Dengue Outbreaks: A Structural Equation Model of a Cross-Sectional Survey
Source: PLoS Negl Trop Dis. 2013 Sep 26;7(9):e2401. doi: 10.1371/journal.pntd.0002401 (PMC3784466; doi:10.1371/journal.pntd.0002401)
Supplement: Text S1 — Questionnaire. (PDF) [file pntd.0002401.s001.pdf]

Ministry of Health Malaysia

University of East Anglia

QUESTIONNAIRES

ID Number [ ][ ][ ]

Days of Outbreak [ ][ ][ ] days

Outbreak Location [ ]

Thank you for agreeing to participate in this research, which is part of a doctoral research study at the School of Medicine, Health Policy & Practice, Faculty of Health, University of East Anglia, Norwich, England.

In this survey, we would like to find out how much you know about what to do in cases of dengue outbreak and how confident you feel you can carry out behaviors that protect you and your family from dengue fever.

Your views are very important to us. During this interview, the interviewer will read all the questions to you. Please note that there are no right or wrong answers. You are not obliged to complete the questionnaire and may discontinue completing the interview at any time. The interview will last approximately 15 minutes.

Your responses and the data obtained during this interview will be kept strictly confidential and will be used for academic purposes only.

If you have any questions or concerns about this survey or being in the study you may also contact me at 6012-323 1980 or a.isa@uea.ac.uk.

Thank you very much for your participation and kind assistance!

Affendi Isa
PhD Researcher
School of Medicine, Health Policy & Practice
Faculty of Health
University of East Anglia
Norwich
NR4 7TJ
England, United Kingdom
Email: a.isa@uea.ac.uk

PART A : RESPONDENT BACKGROUND

INSTRUCTION TO INTERVIEWER **: Please tick (√) the respondent's answer in the provided boxes.**

FOR RESEARCHER USE

1 Gender: ☐ Male ☐ Female — **A1** ☐

2 D.O.B: ☐☐ *Day* ☐☐ *Month* ☐☐☐☐ *Year* — **A2** ☐☐ . ☐

3 Race: ☐ Malay ☐ Chinese ☐ Indian ☐ Others — **A3** ☐

4 Occupation: ☐ Full time ☐ Part time ☐ Housewife ☐ Unemployed / Student — **A4** ☐

5 Marital Status: ☐ Married ☐ Divorcee ☐ Single ☐ Widow / Widower — **A5** ☐

6 Education: ☐ University / College ☐ Primary School ☐ Secondary School ☐ No formal education — **A6** ☐

7 How many people living in the house? ☐☐ — **A7** ☐☐

8 How many children <16 years old living in the house? ☐☐ — **A8** ☐☐

8a How many children study at Primary school? ☐☐ — **A8a** ☐☐

8b How many children study at Secondary school? ☐☐ — **A8b** ☐☐

10 Estimate monthly household income?
☐ Below RM500
☐ RM501 - RM1000
☐ RM1000 - RM2500
☐ RM2500 and above — **A10** ☐

PART B: HEALTH PROMOTION & EDUCATION EXPOSURE

FOR RESEARCHER USE

11 Did you get involved in health promotion activities on dengue within the last 14 days? **B11** ☐

☐ Yes ☐ No *(proceed to question no 10)*

12 What type of health promotion activities did you get invloved within last 14 days?
(more than one answer possible):

☐ Public Lecture ☐ Small Group Discussion
☐ Source Reduction ☐ Demonstration
☐ Individual Advice ☐ Others: ____________________ **B12** ☐

13 How many times did you get involved in above activities within last 14 days?

☐☐☐ times **B13** ☐☐☐

14 Did you receive information on dengue within the last 14 days?

☐ Yes ☐ No *(proceed to Part C)* **B14** ☐

15 What sources of the information on dengue were you exposed to in the last 14 days?
(may have more than one answer):

☐ Public Announcements ☐ Printed Media
☐ Outdoor Media ☐ Radio [Local/National]
☐ TV [Local / National] ☐ Newspaper [Local National] **B15** ☐☐☐

16 How many times were you exposed to the above information within the last 14 days?

☐☐☐ times **B16** ☐☐☐

17 After encountering the health information and promotion on dengue within the last 14 days, how many days did it take you to engage in dengue prevention activities on your own?

☐☐ day(s) **B17** ☐☐

PART C: KNOWLEDGE ON DENGUE

Intruction to Interviewer: Please read the questions clearly and then tick the answers given by the respondents in the provided boxes.

FOR RESEARCHER USE

18 How is dengue fever transmitted to a person?

- ☐ Mosquito bite
- ☐ Blood transmission
- ☐ Airborne
- ☐ Waterborne
- ☐ Don't know / Others

C18 ☐

19 What is the type of mosquito which that dengue fever?

- ☐ Aedes
- ☐ Anopheles
- ☐ Culex
- ☐ Don't know / Others

C19 ☐

20 When is usually Aedes active to bite?

- ☐ 6am - 8am only
- ☐ 6am - 8am & 7pm - 9pm
- ☐ Other times or don't know

C20 ☐

21 What are the signs and symptoms of dengue fever?

- ☐ High fever
- ☐ Chills
- ☐ Headache
- ☐ Eye pain
- ☐ Enlarge lymph nodes
- ☐ Deep muscle and joints pain
- ☐ Loss of appetite
- ☐ Nausea and vomitting
- ☐ Diarrhea
- ☐ Extreme fatigue
- ☐ Others …………….

C21 ☐

22 What steps do you take to prevent dengue fever transmission during the outbreak?

- ☐ Cover tightly all water containers
- ☐ Keep drain free from blockage
- ☐ Change water in plant container
- ☐ Change water in trays under the fridge
- ☐ Bury unused tyres, if any.
- ☐ Adding larvacide in water containers
- ☐ Remove water from flower pot trays
- ☐ Destroy / burn unused containers
- ☐ Place all garbage that can accumulate water into closed bin
- ☐ Level defective floor surfaces that can collect water, if any.
- ☐ Use mosquito repellent and mosquito net when sleeping
- ☐ Others *(please speficy)* ………………………………………………………….

C22 ☐ / ☐

23 What you must do during the biological / chemical prevention activities take place?

- ☐ Allow inspection of mosquito larvae inside and outside the house
- ☐ Allow the Health Authority to put larvacidals in potentially breeding sites
- ☐ Allow the Health Authority to do fogging inside and outside house
- ☐ Open house windows during the fogging process
- ☐ Others *(please speficy)* ………………………………………………………….

C23 ☐ / ☐

PART C: KNOWLEDGE ON DENGUE

Intruction to Interviewer : Please read the questions clearly and then tick the answers given by the respondents in the provided boxes.

FOR RESEARCHER USE

24 What do you do when you get fever during the dengue outbreak?

- ☐ Seek immediate medical attention / treatment
- ☐ Use medication as prescribed after seeing the doctor
- ☐ Others *(please speficy)* ……………………………………………………………….

C24 ☐ / ☐

25 Where you think aedes mosquito usually breeds inside the house?

- ☐ In the tray under the fridge
- ☐ In the water container
- ☐ In the flower pot trays
- ☐ In the opened water tank
- ☐ Others *(please speficy)* ……………………………………………………………….

C25 ☐ / ☐

26 Where you think aedes mosquito usually breeds outside the house?

- ☐ In the flower leaves
- ☐ In the roof gutter
- ☐ In the abandoned tyres
- ☐ In the garbage
- ☐ Others *(please speficy)* ……………………………………………………………….

C26 ☐ / ☐

PART D : BEHAVIORAL SELF-EFFICACY

Strength of Self-Efficacy

Instruction: Please rate respondents confidence level in the provided boxes by choosing number 1 to 10 to the statements below according to the following scales.

0	1	2	3	4	5	6	7	8	9	10
Not at all confident					***Moderately confident***					***Extremely confident***

		**FOR RESEARCHER USE**	
**27** I can change the water in plant pot trays every week.	☐	**D27**	☐
**28** I can clean the drain from blockage every 7 days	☐	**D28**	☐
**29** I can always cover tightly all water containers inside and outside house	☐	**D29**	☐
**30** I can convince my children to always put all garbage into closed bin	☐	**D30**	☐
**31** I can go or bring my family member to see a doctor immediately when I or they fall sick	☐	**D31**	☐
**32** I can change the water in the container under the fridge every week	☐	**D32**	☐
**33** I can allow health authority to fog and inspect my house at anytime	☐	**D33**	☐
**34** I can convince my neighbours to do weekly search and destroy any potential aedes breeding sites outside the house	☐	**D34**	☐
**35** I can always put larvacide in all water containers inside my house	☐	**D35**	☐
**36** I can always ask my family to sleep in mosquito net every night	☐	**D36**	☐

PART D : BEHAVIORAL SELF-EFFICACY

Level of Self-Efficacy

In this section, a basic scene will be described, and I will ask you three questions. The scence will be repeated two more times, but some details will change each time, making the situation more difficult. For each of the three questions that follow the scences, you will be asked to rate how confident you are that you could do certain things if you were to find yourself in that situation today. Rate the degree of confidence that you are feeling ***RIGHT NOW*** *by recording a number from 1 to 10 on the scale given after each question.*

Scene No 1

There is no dengue outbreak in your village, but one of your family members has high fever. You are pretty busy at work during the weekdays. You still have 10 minutes a week to search and destroy potential aedes breeding sites inside and outside your house. One day, you find a water container that is full of mosquito larvae behind your neighbour's house.

Question 1 (D37)

How confident are you that you could bring up the issue of removing the water container to your neighbour in this situation?

0	1	2	3	4	5	6	7	8	9	10
Not at all confident					***Moderately confident***					***Extremely confident***

Question 2 (D38)

How confident are you that you could convince you neighbour to do 10 minutes weekly search and destroy to any potential aedes breeding sites?

0	1	2	3	4	5	6	7	8	9	10
Not at all confident					***Moderately confident***					***Extremely confident***

Question 3 (D39)

If your neighbour refused to destroy the container, how confident are you that you report him to the local Health Authority?

0	1	2	3	4	5	6	7	8	9	10
Not at all confident					***Moderately confident***					***Extremely confident***

PART D : BEHAVIORAL SELF-EFFICACY

Level of Self-Efficacy

Scene No 2

There is no dengue outbreak in your village, but there is an uncontrolled outbreak at a nearby village with several cases reported. One of your family members also has high fever. You are pretty busy at work during the weekdays. You still have 10 minutes a week to search and destroy to potential aedes breeding sites inside and outside your house. One day, you find a water container that is full of mosquito larvae behind your neighbour's house.

Question 1 (D40)

How confident are you that you could bring up the issue of removing the water container to your neighbour in this situation?

0	1	2	3	4	5	6	7	8	9	10
Not at all confident					***Moderately confident***					***Extremely confident***

Question 2 (D41)

How confident are you that you could convince you neighbour to do 10 minutes weekly search and destroy to any potential aedes breeding sites?

0	1	2	3	4	5	6	7	8	9	10
Not at all confident					***Moderately confident***					***Extremely confident***

Question 3 (D42)

If your neighbour refused to destroy the container, how confident are you that you report him to the local Health Authority?

0	1	2	3	4	5	6	7	8	9	10
Not at all confident					***Moderately confident***					***Extremely confident***

PART D : BEHAVIORAL SELF-EFFICACY

Level of Self-Efficacy

Scene No 3

There is a dengue outbreak in your village with many cases reported and several deaths. One of your family members is also suspected to have dengue fever and has been hospitalised. The outbreak is uncontrolled and prolonged for more than 14 days. You are pretty busy at work during the weekdays. You still have 10 minutes a week to search and destroy potential aedes breeding sites inside and outside your house. One day, you find several water containers with full of mosquito larvae behind several of your neighbour's houses.

Question 1 (D43)

How confident are you that you could bring up the issue of removing the water container to your neighbour in this situation?

0	1	2	3	4	5	6	7	8	9	10
Not at all confident					***Moderately confident***					***Extremely confident***

Question 2 (D44)

How confident are you that you could convince you neighbour to do 10 minutes weekly search and destroy to any potential aedes breeding sites?

0	1	2	3	4	5	6	7	8	9	10
Not at all confident					***Moderately confident***					***Extremely confident***

Question 3 (D45)

If your neighbour refused to destroy the container, how confident are you that you report him to the local Health Authority?

0	1	2	3	4	5	6	7	8	9	10
Not at all confident					***Moderately confident***					***Extremely confident***
