# Supplementary material for: Mediational Effects of Self-Efficacy Dimensions in the Relationship between Knowledge of Dengue and Dengue Preventive Behaviour with Respect to Control of Dengue Outbreaks: A Structural Equation Model of a Cross-Sectional Survey
Source: PLoS Negl Trop Dis. 2013 Sep 26;7(9):e2401. doi: 10.1371/journal.pntd.0002401 (PMC3784466; doi:10.1371/journal.pntd.0002401)
Supplement: Text S2 — Additional tables. (PDF) [file pntd.0002401.s002.pdf]

Table S1: Scores for Involvement in Health Promotion Activities

**Days of Involvement**	**Mark**
0 to 1	0
1 to 2	1
3 to 5	2
6 to 10	3
≥ 11	4

Table S2**.** Health Promotion Exposures Calculation Based on Kriska's MAQ (1997)

**Calculation**	**Domain**	**Maximum Marks**
A	***Number of activities involved***<br>Questionnaires No 11 X (No 12a to No 12e) X No 13	60
B	***Number of information received***<br>Questionnaires No 14 X (No 15a to No 15f)	20
C	***Score of recent behaviour compliance***<br>Questionnaires No 16 X No 17	20
	**TOTAL**	**100**

Table S3. Indication of Knowledge Level Based on Bloom's Taxonomy

Score %	Knowledge Level
80 – 100	Excellent (Application)
50 – 79.9	Good (Comprehension)
0 – 49.9	Poor (Knowledge Level)

Table S4. Level and Strength of Self-Efficacy Categorization

**Mean Scales**	**Level & Strength of Self-Efficacy**
9 - 10	Excellent
7 – 8.99	Good
5 – 6.99	Moderate
3 – 4.99	Low
0 – 2.99	Very Low / Poor
